# Patient satisfaction with preoperative nursing care and its associated factors in surgical procedures, 2023: a cross-sectional study

**DOI:** 10.1186/s12912-024-01881-5

**Published:** 2024-04-08

**Authors:** Bizuayehu Atinafu Ataro, Temesgen Geta, Eshetu Elfios Endirias, Christian Kebede Gadabo, Getachew Nigussie Bolado

**Affiliations:** 1 Adult Health Nursing, School of Nursing, College of Health Science and Medicine, Wolaita Sodo University, Sodo, Ethiopia; 2 Maternity and Child Health Nursing, School of Nursing, College of Health Science and Medicine, Wolaita Sodo University, Sodo, Ethiopia; 3 Pediatrics and Child Health Nursing, School of Nursing, College of Health Science and Medicine, Wolaita Sodo University, Sodo, Ethiopia

**Keywords:** Preoperative nursing care, Patient satisfaction, Surgical procedure, Associated factors

## Abstract

**Background:**

To enhance patient satisfaction, nurses engaged in preoperative care must possess a comprehensive understanding of the most up-to-date evidence. However, there is a notable dearth of relevant information regarding the current status of preoperative care satisfaction and its impact, despite a significant rise in the number of patients seeking surgical intervention with complex medical requirements.

**Objective:**

To assess patient satisfaction with preoperative nursing care and its associated factors in surgical procedures of, 2023.

**Methods:**

A cross-sectional study was conducted, and the data was collected from the randomly selected 468 patients who had undergone surgery during the study period. The collected data was entered into Epidata version 3.1 and analyzed using SPSS version 25 software.

**Results:**

The complete participation and response of 468 participants resulted in a response rate of 100%. Overall patient satisfaction with preoperative nursing care was 79.5%. Sex (Adjusted odds ratio (AOR): 1.14 (95% confidence interval (CI): 0.21–2.91)), payment status for treatment (AOR: 1.45 (95% CI: 0.66–2.97)), preoperative fear and anxiety (AOR: 1.01, 95% CI: 0.49–2.13)), patient expectations (AOR: 3.39, 95% CI: 2.17–7.11)), and preoperative education (AOR: 1.148, 95% CI: 0.54–2.86)) exhibited significant associations with patient satisfaction with preoperative nursing care.

**Conclusion:**

It is important to exercise caution when interpreting the level of preoperative nursing care satisfaction in this study. The significance of preoperative nursing care satisfaction lies in its reflection of healthcare quality, as even minor deficiencies in preoperative care can potentially lead to life-threatening complications, including mortality. Therefore, prioritizing the improvement of healthcare quality is essential to enhance patient satisfaction.

## Background

Preoperative care encompasses the provisions given prior to surgery, wherein the patient’s unique requirements are considered to undertake physical and psychological preparations in anticipation of the procedure [1]. This phase commences upon the patient’s admission to the hospital or surgical facility and extends until the commencement of the actual procedure [1–4]. The primary emphasis in preoperative preparation should lie in the advancement of techniques aimed at mitigating the emotional distress experienced by surgical patients [5]. In this context, nurses play a crucial role in formulating, developing, expanding, and implementing interventions and modifications [5, 6].

The primary goal of a healthcare system is to ensure the provision of medical care that is of the utmost quality and safety [7]. In this context, patient safety has emerged as a paramount concern and is currently placed at the forefront of priorities [8, 9]. A systematic review conducted in Saudi Arabia and Turkey concluded that preoperative nursing assessment plays a vital role in mitigating preoperative complications by alleviating anxiety and enhancing patients’ understanding of the surgical procedure. This, in turn, has a substantial positive impact on patient satisfaction [10, 11]. The review also emphasized the necessity of nurses receiving proper training and education in preoperative assessment, as the absence of adequately trained nursing staff elevates patient anxiety levels and renders them susceptible to potential complications [2, 10].

Patient satisfaction is defined as a subjective reaction to the context, process, and result of the service experience one has received [12, 13]. The measurement of quality is closely linked to the satisfaction levels expressed by patients regarding the care they have received [14, 15]. Both the practice environment and the personal characteristics of nurses serve as significant indicators of the quality of patient care [16]. Enhancing working conditions and achieving improved patient outcomes, including reduced mortality rates, are facilitated by a positive relationship between the work environment attributes of nurses and their levels of proficiency and personal capabilities [17]. Additionally, various aspects of the workplace, such as the physical setting, working hours, and the level of fatigue among nursing staff, have been found to influence the safety and quality of patient care [18].

Comprehensive nursing interventions should be implemented throughout the entire perioperative phase to prevent complications and adverse events in the surgical domain [19]. Although the impact of perioperative nursing interventions on patient health outcomes may not be fully comprehended, it is substantial in its significance [20]. Through the provision of care during the postoperative period, nurses can effectively mitigate the occurrence of adverse events, even though certain studies have identified nurses’ workload and time constraints as predominant barriers to effective nurse-patient communication [21–24]. Preoperative nursing assessment plays a pivotal role in delineating and discerning the patient’s risk factors throughout their perioperative care, extending beyond the confines of the surgical procedure itself [25, 26].

To optimize patient care and enhance postoperative outcomes, it is imperative for nurses engaged in patient assessment and preoperative care to possess comprehensive knowledge and understanding of the latest research in this field [27]. Throughout the preoperative phase, nurses provided comfort, guidance, and rehabilitation to the patients. However, they failed to involve the patients in their treatment [28, 29]. An unfortunate number of patients endured minor injuries due to improper utilization of theater equipment, such as diathermy devices, along with inadequate implementation of safety precautions by the nursing staff during the surgical procedure [28, 30]. Furthermore, patients were left feeling bewildered and unsettled due to the nurses’ deficient communication [28, 31].

The perioperative environment possesses distinctive characteristics, encompassing intricate clinical care delivered by specialized teams, substantial costs, utilization of advanced technologies, and a vast array of challenging-to-manage resources [30, 32]. These factors can contribute to the development of highly intricate settings prone to adverse events concerning patient safety [32, 33]. Medication errors, omissions, patient misidentification, and surgical site misidentification are among the various types of mistakes that can occur during surgical procedures [34]. Birmingham-based research showcased that reducing waiting times, enhancing patient satisfaction, and upholding the efficacy of clinical services were the outcomes of evaluating patient load and the delivery system within the clinic [35, 36]. To optimize patient satisfaction, nurses involved in preoperative care must possess up-to-date knowledge and understanding of the most recent research [27]. Despite the significant increase in the number of patients requiring surgery, with complex medical needs, a scarcity of pertinent data exists regarding the satisfaction levels and impacts associated with preoperative care.

Studies conducted in Ethiopia showed varying levels of patient satisfaction with preoperative nursing care in surgical procedures. The cross-sectional study carried out in Addis Ababa, Western Amhara referral hospitals, University of Gondar Comprehensive Specialized Hospital, East Amhara referral hospitals and Gamo and Gofa zone showed that the patient satisfaction with preoperative care ranges from 36.6 to 84% [12, 37–40]. According to the study conducted at Sohag University, the overall satisfaction score of patients who underwent surgery was determined to be 61.9% [41].

Various factors play key roles in influencing patient satisfaction with preoperative nursing care, both related to the hospital and nursing environment (such as ward/unit dynamics, length of hospitalization, surgical specialization, waiting times, nurse responsiveness), patient and family characteristics (including financial status, prior hospitalizations, service expectations, health conditions, procedure types, complications, discharge plans, anxiety levels, illness duration, family size), and preoperative education can seriously influence satisfaction levels of patients with preoperative nursing care. Additionally, sociodemographic factors like gender, age, income, residence, marital status, religion, ethnicity, education level, and occupation may also significantly impact patient satisfaction [1, 10, 12, 32, 37–42].

Enhancing patient satisfaction with preoperative nursing care is vital for patient-centered healthcare. This study investigates the factors influencing patient satisfaction in surgical procedures, aiming to improve care quality. By identifying areas for enhancement, the research informs healthcare practices, potentially leading to better patient experiences and outcomes. Contributing to the existing literature, this contemporary study provides updated insights into patient preferences, guiding efforts toward optimized preoperative care delivery and improved surgical outcomes. This research can also pave the way for advancements in patient-centered care approaches and potentially lead to positive impacts on healthcare outcomes and patient experiences in surgical settings.

Most of the previous research conducted in Ethiopia has primarily focused on evaluating patient satisfaction with the overall hospital services. However, this particular study honed in on specifically examining the satisfaction levels of preoperative nursing care services. This focus was chosen due to the profound impact that such care has on surgical outcomes and subsequent postoperative recovery. Notably, this study stands as the first of its kind within our study area; as far as we know, no prior study of this nature has been conducted. It is also worth noting that while some previous studies had utilized nurses as study participants, this study appropriately selected patients, as they possess indispensable insights into the quality of nursing care and ultimately determine the level of satisfaction experienced. Additionally, this study introduced previously unstudied variables, such as patient flow per shift and nurses’ willingness to listen and respond to questions, which hold the potential for significant associations with satisfaction levels regarding preoperative nursing care services. Therefore, this study aimed to comprehensively assess patient satisfaction with preoperative nursing care and its associated factors in surgical procedures.

## Methods and materials

### Study area and period

This study was carried out in the Wolaita Zone, located 329 km away from Addis Ababa, the capital of Ethiopia. Currently, Wolaita Sodo serves as the capital city of southern Ethiopia. Known for its high population density, the zone boasts 290 individuals per square kilometer, making it one of the most densely populated regions in the country. According to the 2021 population projection by the Central Statistical Agency of Ethiopia, the Wolaita Zone is home to a total population of 6,142,063 people residing within an area of 4,208.64 square kilometers (1,624.96 sq. mi). Within this zone, there are nine public hospitals, with Wolaita Sodo University Comprehensive Specialized Hospital being the sole specialized healthcare facility. The hospital provides a broad range of surgical services spanning multiple departments, including general surgery, orthopedic surgery, urologic surgery, obstetrics and gynecologic surgery, and maxillofacial surgery. The study was conducted from July 15 to July 30, 2023.

#### **Study design:**

Facility-based cross-sectional study was employed because it allows for the exploration of relationships between variables at a specific moment, providing valuable insights into the prevalence of patient satisfaction and associated factors concurrently.

### Populations

#### **Source population:**

All surgical patients who have undergone surgery.

#### **The study sample:**

All surgical patients that are available during a study period.

### Eligibility

#### **Inclusion criteria:**

All adult patients aged ≥ 18 years who have undergone surgery and have been admitted to a surgical, obstetrics/gynecology ward, ophthalmic, orthopedic, or other department were included.

#### **Exclusion criteria:**

Patients who sought treatment as outpatients, individuals who were severely ill and unconscious, as well as patients with known mental health issues, were excluded from the study.

### Sample size determination and procedure

The sample size was determined using a formula for a single population proportion, taking into account the following assumptions: a prevalence of 52.75% for patient satisfaction with nursing care in Eastern Ethiopia [25], a confidence level of 95%, a margin of error of 5%, a nonresponse rate of 10% as follows:

$${{{\rm{n = }}\left( {{\rm{Z \alpha /2}}} \right){\rm{2}}\,{\rm{x}}\,{\rm{P }}\left( {{\rm{1 - P}}} \right)} \over {{\rm{d2}}}}$$

**where**:

n- The minimum sample size required.

P- Prevalence of satisfaction with preoperative nursing care.

d- Margin of error.

Z𝛼/2- Standard normal distribution at 95% confidence level

$${{{\rm{n = }}\left( {{\rm{1}}{\rm{.96}}} \right){\rm{2\, X0}}{\rm{.5275 }}\left( {{\rm{1 - 0}}{\rm{.5275}}} \right){\rm{ = 425}}} \over {{{\left( {{\rm{0}}{\rm{.05}}} \right)}^{\rm{2}}}}}$$

After accounting for a 10% contingency for potential non-response, the final sample size for this study amounted to 468 subjects.

### Study variables

#### **Dependent variable:**

Patients’ satisfaction.

#### **Independent variables:**

Sociodemographic variables (sex, age, monthly income, residence, marital status, religion, ethnicity, educational, occupational status); Hospital and nurse-related variables (ward/unit, length of hospital stay, surgical specialty, surgery waiting time, patient flow per shift, nurses’ willingness to listen and respond to questions); Patient and family factors (payment status, previous admission, patient service expectations, co-morbidity, surgery type, complications, discharge destination, preoperative fear and anxiety, duration of the illness, family size), and Preoperative education.

### Data collection tools and procedures

The data was collected through a meticulously tested, structured, interview-administered questionnaire originally written in English and then translated into the local language, Wolaitigna, to ensure accessibility and accurate comprehension among the participants. The questionnaire was divided into six sections and was obtained from previous studies conducted in Ethiopia and other locations internationally [12, 13, 31, 39]. The first part of the questionnaire contains the sociodemographic characteristics of the patients. The second part contains institution- or health facility-related variables affecting patients’ preoperative nursing care services. Items in the third and fourth sections assessed the nurse-related factors and patient- and family-related variables influencing patients’ preoperative nursing care services, respectively. One of the patient-related factors was preoperative fear and anxiety and it was measured by tools adapted from previous studies conducted in Ethiopia and Iraq [43, 44]. The fifth part of the question contains items used to measure preoperative education containing 16 questions [12]. The final part contains items to measure the level of preoperative nursing care satisfaction among nurses. The instruments utilized to assess patient satisfaction with preoperative nursing care comprised a set of 22 Likert-scale questions. Each question was rated on a scale from 1, indicating “very unsatisfied,” to 5, indicating “very satisfied”. This tool was valid in Ethiopia and had internal consistency with Cronbach’s alpha of 0.96. The overall patient satisfaction with preoperative nursing care in surgical procedures was classified into two categories: satisfied and unsatisfied [12, 31, 37].. A team of four nursing professionals who held BSc degrees was specifically assigned to take on the role of data collectors. They were closely supervised by two BSc-qualified nurse professionals throughout the study, who were selected from Sodo Health Center.

### Data processing and analysis

The collected data were cleaned, coded, and entered using Epidata software and exported into Statistical Package for the Social Sciences (SPSS) Version 26 to facilitate analysis. To explore the relationship between the dependent and independent variables, both bivariable and multivariable logistic regression techniques were utilized. In the bivariable logistic regression model, all independent variables with a p-value less than 0.25 were subsequently entered into the multivariable logistic regression model. The evaluation of significance relied on the adjusted odds ratio (AOR), accompanied by a 95% confidence interval (CI) and a p-value less than 0.05, allowing for meaningful interpretation of the obtained associations. Descriptive statistics, such as tables, graphs, frequencies, and percentages, were employed to provide an overview of the characteristics observed within the sample.

### Data quality control

A preliminary assessment, commonly referred to as a pilot study, of the questionnaire, took place at Grace Primary Hospital, which lies outside the scope of the target hospitals. This pre-test was conducted on a subset of the sample size, comprising 5%, a week before the commencement of the actual data collection period. Based on the outcomes of the pre-test, necessary modifications were made to address issues such as unclear questions, typographical errors, and ambiguous wording. Furthermore, the reliability of the Likert-scale items was assessed using Cronbach’s alpha, yielding a coefficient of 0.82. To ensure proficient data collection, a comprehensive one-day training session was provided to the data collectors, encompassing instructions on both the data collection tool and the collection process itself. The principal investigator oversaw the data collection process and monitored its completeness, accuracy, and consistency daily. To enhance data integrity, a double-entry method was employed, involving two separate data clerks who independently entered the collected data into SPSS. The consistency of the entered data was cross-verified by comparing the two versions of the data to identify any discrepancies.

## Results

### Socio-demographic characteristics of the participants

The response rate for this study was an impressive 100%. Out of the total of 468 respondents, the majority were female (55.1%), and the mean age of the participants was 34 years with a standard deviation of 8.9. Notably, a significant proportion (21.6%) fell within the age bracket of 25 to 34 years. Among the respondents, 210 (44.9%) resided in urban areas, while 258 (55.1%) hailed from rural regions. Regarding marital status, the majority (68.6%) were married, and adherents of the Protestant faith constituted more than 50% of the participants. Approximately 60% of the respondents were illiterate, and 138 (29.5%) identified themselves as farmers. Furthermore, 131 (28.0%) were engaged in the role of housewives, and 107 (22.9%) were students. Out of the total 468 respondents, 223 (47.6%) reported earning less than 1000 ETB per month (Table 1).

**Table 1:** Sociodemographic characteristics of patient patients undergoing surgical procedures at Wolaita Sodo University Comprehensive Specialized Hospital, Southern Ethiopia, 2023 (n = 468)

Variable		Frequency (n)	Percent (%)
Sex	Male	210	44.9
	Female	258	55.1
Age	18-24	51	10.9
	25-34	101	21.6
	35-44	94	20.1
	45-54	91	19.4
	55-64	54	11.5
	65 and above	77	16.5
Marital status	Married	321	68.6
	Single	133	28.4
	Others*	8	3.0
Residence	Rural	258	55.1
	Urban	210	44.9
Religion	Protestant	238	50.9
	Orthodox	138	29.5
	Muslim	52	11.1
	Catholic	28	6.0
	Others	12	2.6
Ethnicity	Wolaita	311	66.5
	Gamo and Gofa	49	10.5
	Dawro	40	8.5
	Tembaro	37	7.9
	Others	31	6.6
Educational status	Unable to write and read	269	57.5
	Able to read and write	75	16.0
	Primary school completed	63	13.5
	Secondary school completed	19	4.1
	Certificate and above	42	9.0
Occupational status	Farmer	138	29.5
	Housewife	131	28.0
	Merchant	66	14.1
	Student	107	22.9
	Daily worker	26	5.6
Monthly income	Lower than 1000 ETB	223	47.6
	1001 to 2500 ETB	14	3.0
	2501 to 3999 ETB	11	2.4
	more than 4000 ETB	84	17.9
	No income/ family dependent	136	29.1

Others* = widowed and divorced

### Patient satisfaction with preoperative nursing care

**Fig. 1:**
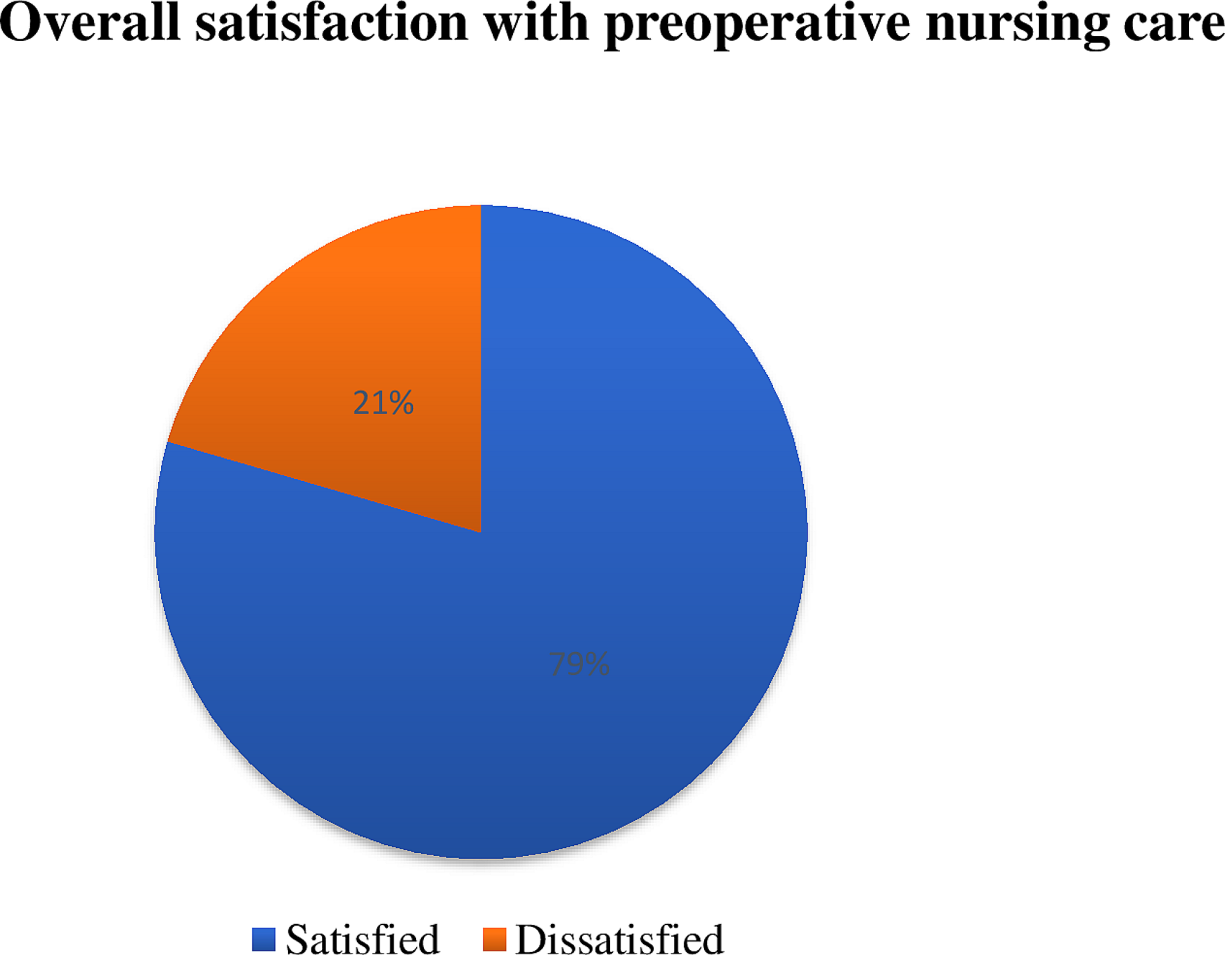
Patient satisfaction with preoperative nursing care in surgical procedures at Wolaita Sodo University Comprehensive Specialized Hospital

The overall satisfaction with preoperative nursing care among patients who have undergone surgical procedures at Wolaita Sodo University Comprehensive Specialized Hospital was 79.5% (75.4–83.6) (Fig. 1).

### Variables influencing patient satisfaction with preoperative nursing care

#### Hospital and nurse-related variables

Among the participants, a substantial majority (84.8%) were admitted to the surgical unit of the hospital, highlighting the prevalence of surgical cases in the study sample. In terms of the duration of hospital stay, 289 (61.8%) reported a stay of less than seven days, indicating relatively shorter periods of hospitalization. When it came to interactions with surgeons, the participants disclosed that 151 (32.2%) had contact with surgeons specializing in general surgery, while 129 (27.6%) had contact with surgeons specializing in traumatology. Regarding the waiting time for surgery, more than half of the participants (53.2%) indicated a waiting period of less than one month. Moreover, a majority of the participants (55.3%) acknowledged that there was a high number of patient or a high patient flow during their waiting period, suggesting the burden on the healthcare system. Disturbingly, 277 (59.2%) of the participants reported dissatisfaction with the nurses’ willingness to listen and respond to their concerns, indicating poor communication and responsiveness on the part of the nursing staff (Table 2).

**Table 2:** Hospital- and nurse-related factors affecting patient satisfaction with preoperative nursing care in surgical procedures at Wolaita Sodo University Comprehensive Specialized Hospital, Southern Ethiopia, 2023 (n = 468)

Variables		Frequency (n)	Percentage (%)
Ward/unit	Surgical	397	84.8
	OBS/GYN	22	4.7
	Orthopedic	44	9.4
	Ophthalmic	5	1.1
Length of hospital stay	Lower than 7 days	289	61.8
	Between 8 to 14 days	40	8.5
	More than 15 days	6	1.3
	Not stated clearly	133	28.4
Surgical specialty	General surgery	151	32.2
	Traumatology	129	27.6
	Gynecology	71	15.2
	Thoracic surgery	52	11.1
	Vascular surgery	21	4.5
	Neurosurgery	24	5.2
	Others	20	4.2
Surgery waiting time	Less than 1 month	249	53.2
	Less than 3 months	99	21.1
	3–6 months	78	16.7
	More than 6 months	42	9.0
Patient-flow per shift	Low	209	44.7
	High	259	55.3
Nurses’ willingness to listen and respond questions	Good	191	40.8
	Poor	277	59.2

### Patient and family variables

Among the respondents who participated in this study, a significant proportion (61.3%) revealed that they had fewer than three family members, while 148 (31.6%) reported having four to six family members. More than half of the participants (53.4%) reported receiving free-of-charge treatment from the hospital, indicating a reliance on the hospital’s financial support. Additionally, a considerable number of respondents (63.7%) recalled previous admissions for various health issues. Similarly, 171 (63.5%) of the patients reported having co-morbidities during their initial diagnosis, further complicating their healthcare journey. A substantial proportion of the participants (83.3%) experienced complications related to their current surgery, with pain being the most prevalent complication, affecting 324 (83.1%) of those experiencing complications. The majority of the participants (40.2%) reported that their illness had persisted for several days before undergoing surgery. Abdominal surgery was the most common surgical procedure among the participants, accounting for 119 (25.4%) cases. As for the anticipated discharge destination, 301 (64.3%) participants stated that they would be returning home upon discharge, emphasizing the preference for familiar surroundings. Unsurprisingly, preoperative fear and anxiety were prevalent among the participants, with 373 (79.9%) reporting experiencing high fear and anxiety. Moreover, a significant majority (78.6%) had high service expectations from the hospital, indicating the importance of quality care and support during the preoperative period (Table 3).

**Table 3:** Patient and family-related factors affecting patient satisfaction with preoperative nursing care in surgical procedures at Wolaita Sodo University Comprehensive Specialized Hospital, Southern Ethiopia, 2023 (n = 468)

Variables		Frequency (n)	Percentage (%)
Family size	Lower than 3 members	287	61.3
	4 to 6 members	148	31.6
	Not clearly stated	33	7.1
Payment status for treatment	Free	250	53.4
	Paid	218	46.6
Previous admission	Yes	298	63.7
	No	170	36.3
Co-morbidity	Yes	171	63.5
	No	297	36.5
Surgery type	Cardiothoracic	37	7.9
	ENT	54	11.5
	Neurology	67	14.3
	Abdominal	119	25.4
	Urology	74	15.8
	Gynecology	50	10.9
	Orthopedics	51	10.9
	Others	16	3.5
Complications	Yes	390	83.3
	No	78	16.7
Complication type	Pain	324	83.1
	Bleeding	38	9.7
	Wound infection	17	4.4
	Others	11	2.8
Discharge destination	Home	301	64.3
	Nursing home	167	35.7
Preoperative fear and anxiety	Yes	373	79.9
	No	95	20.1
Duration of the illness	In days	188	40.2
	In months	166	35.5
	In years	114	24.3
Patient expectations	High	368	78.6
	Medium	61	13.0
	Low	39	8.4

### Patient satisfaction with preoperative education

**Fig. 2:**
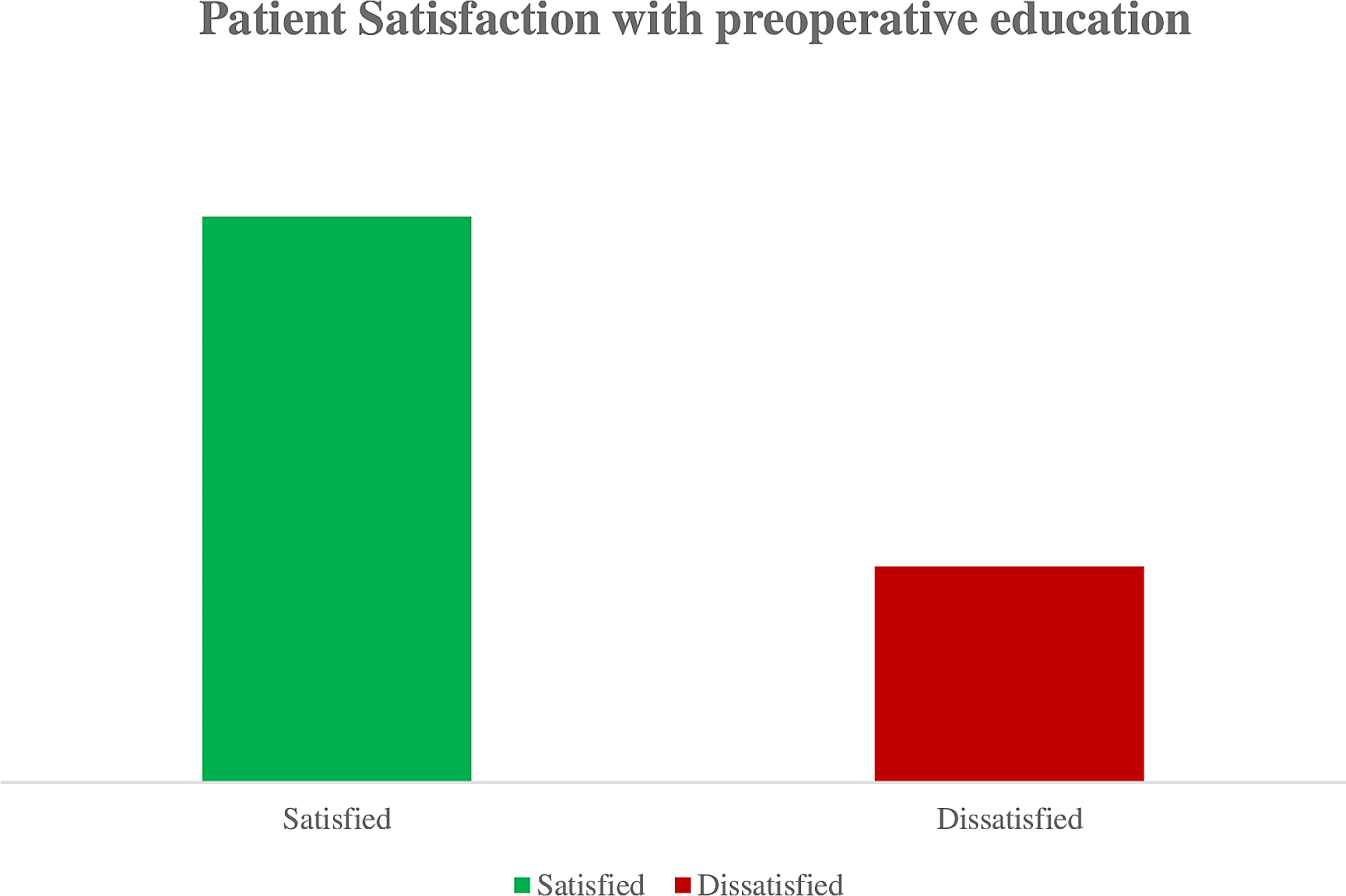
Overall patient satisfaction with preoperative education on surgical procedures at Wolaita Sodo University Comprehensive Specialized Hospital

The overall patient satisfaction with preoperative education on surgical procedures was 79.5% (Fig. 2).

### Factors associated with satisfaction with preoperative nursing care

Sex, age, educational status, monthly income, length of hospital stays, surgery waiting time, nurses’ willingness to listen and respond, payment status for treatment, complications, duration of illness, preoperative fear and anxiety, patient expectations, and preoperative education were all evaluated as potential factors in the bivariable logistic regression analysis (p < 0.25) to determine their association with patient satisfaction with preoperative nursing care. In the multivariable logistic regression, it was found that sex, payment status for treatment, preoperative fear and anxiety, patient expectations, and preoperative education exhibited significant associations with patient satisfaction with preoperative nursing care (p < 0.05). Male patients were found to be 1.14 times more likely to report satisfaction with preoperative nursing care compared to female patients (AOR: 1.14 (95% CI: 0.21–2.91)). Patients who received free treatment were found to be 1.45 times more likely to express satisfaction with preoperative nursing care compared to those who had to pay for their treatment (AOR: 1.45 (95% CI: 0.66–2.97)). Participants who did not experience preoperative fear and anxiety were found to be 1.01 times more likely to report satisfaction with preoperative nursing care compared to those who did have preoperative fear and anxiety (AOR: 1.01, 95% CI: 0.49–2.13). Patients who had low expectations of hospital services were found to be 3.39 times more likely to express satisfaction with preoperative nursing care compared to those who had high service expectations from the hospital (AOR: 3.39, 95% CI: 2.17–7.11). Participants who received preoperative education from nurses were 1.15 times more likely to be satisfied with preoperative nursing care compared to those who did not receive such education from nurses (AOR: 1.148, 95% CI: 0.54–2.86) (Table 4).

**Table 4:** Bivariable and multivariable binary logistic regression analysis on factors associated with patient satisfaction with preoperative nursing care in surgical procedures at Wolaita Sodo University Comprehensive Specialized Hospital, Southern Ethiopia, 2023 (n = 468)

Variable		Preoperative nursing care satisfaction		COR	AOR (95% CI)
		Good	Poor		
Sex	Male	150	60	1.83	1.14 (0.21-2.91)
	Female	149	109	1	1
Age (in years)	18-24	30	21	1.63	1.45 (0.63-3.21)
	25-34	59	42	1.6	0.94 (0.54-1.61)
	35-44	50	44	1.29	0.923 (0.63-1.68)
	45-54	43	48	1.02	0.573 (0.15-2.21)
	55-64	23	31	0.84	0.27 (0.05-1.45)
	≥65	36	41	1	1
Educational status	Unable to write & read	139	130	1.18	0.824 (0.41-1.66)
	Able to read and write	44	31	1.56	1.201 (0.58-2.27)
	Primary school	36	27	1.47	1.04(0.012-1.17
	Secondary school	8	11	0.8	0.41 (0.30-1.54)
	Certificate and above	20	22	1	1
Monthly income (ETB)	≤ 1000	82	141	0.49	0.77 (0.05-1.19)
	1001- 2500	5	9	0.46	1.01 (0.61-1.21)
	2501 - 3999	6	5	1.01	1.56 (0.98-2.33)
	≥4000	45	39	0.97	0.609 (0.29-1.44)
	No income/ dependent	74	62	1	1
Length of hospital stay	Lower than 7 days	197	92	2.31	3.01 (1.93-3.73)
	Between 8 to 14 days	21	19	1.19	1.56 (1.12-3.0)
	More than 15 days	4	2	2.16	1.49 (0.83-3.12)
	Not stated clearly	64	69	1	1
Surgery waiting time	Less than 1 month	169	80	1.17	0.77 (0.41-1.39)
	Less than 3 months	64	35	1.02	0.72 (0.55-1.46)
	3–6 months	41	37	0.62	1.13 (0.32-2.87)
	More than 6 months	27	15	1	1
Nurses’ willingness to listen and respond questions	Good	86	105	1.17	0.92 (0.64-1.6)
	Poor	114	163	1	1
Payment status for treatment	Free	170	80	1.87	1.45 (0.66-2.97)
	Paid	116	102	1	1
Complication	Yes	186	204	1	1
	No	47	31	1.66	1.93 (1.07-4.11)
Duration of illness	In days	101	87	1.78	1.34 (0.99-2.01)
	In months	89	77	1.77	0.99 (0.47-2.01)
	In years	45	69	1	1
Preoperative fear and anxiety	Yes	178	195	1	1
	No	55	40	1.51	1.01 (0.49-2.13)
Patient expectations	High	177	191	1	1
	Medium	35	26	1.45	1.141 (0.53-2.01)
	Low	31	8	4.18	3.39 (2.17-7.11)
Preoperative education	Yes	211	96	1.38	1.15 (0.54-2.86)
	No	99	62	1	1

## Discussion

The primary objective of this study was to determine the level of patient satisfaction with preoperative nursing care at Wolaita Sodo University Comprehensive Specialized Hospital. Furthermore, the study sought to identify factors significantly associated with patient satisfaction with preoperative nursing care. Consequently, the findings of this study demonstrated that the level of patient satisfaction with perioperative nursing care was 79.5%.

This finding was lower when compared with the previous studies conducted at the University of Gondar Teaching Hospital (98.1%) [31] and Public hospitals in Addis Ababa (84%) [12]. This disparity can potentially be attributed to various factors, including differences in patient variables such as sociodemographic characteristics, variations in hospital settings, potential inadequacies in the provision of preoperative education and care within the hospitals examined in this study, an increased influx of patients, heightened health-seeking behaviors among individuals, as well as elevated patient expectations regarding the quality of services rendered by the hospitals.

Nevertheless, it is noteworthy that the current finding exhibited a higher level of satisfaction when compared with previous studies conducted at Sohag University (61.9%) [41], Western Amhara referral hospitals (68.7%) [37], Gondar University Comprehensive Specialized Hospital (74%) [39], East Amhara referral hospitals (38.5%) [40], and Gamo and Gofa zones (36.6%) [38]. This discrepancy could potentially be attributed to various factors such as differences in the time gaps between the studies, variations in the study participants (for example, the study in East Amhara referral hospitals focused on nurses instead of patients), discrepancies in the services assessed (for instance, the study in the University of Gondar Comprehensive Specialized Hospital solely evaluated satisfaction related to anesthesia services), as well as variances in the perception of the services provided by the patients themselves and the methodologies employed in the studies.

The sex of the patient was significantly associated with patient satisfaction with preoperative nursing care. Male patients were found to be 1.14 times more likely to report satisfaction with preoperative nursing care compared to female patients. This was in line with the study conducted in Barcelona, Spain, [13] which, strengthens that men patients were more satisfied with preoperative nursing care than women. This finding may be attributed to the fact that women reported experiencing more challenges with hospital care when compared to men. This disparity could potentially arise from the fact that female patients place greater emphasis on their health and often assume the role of evaluators and even administrators of care practices, not just for themselves but also for other family members [22].

Similarly, payment status for treatment had a significant association with patient satisfaction with preoperative nursing care. Patients who received free treatment were found to be 1.45 times more likely to express satisfaction with preoperative nursing care compared to those who had to pay for their treatment. This could be because patients who receive treatment for free may view it as a gesture of kindness or support, which can enhance their overall experience and level of satisfaction with the preoperative care they receive. Furthermore, patients who do not have to pay for their medical needs may feel less stressed and anxious about the expense, which frees them up to concentrate more on the quality of nursing care they receive. Furthermore, patients who receive free treatment could feel appreciative of the hospital or healthcare system, which could affect how they feel about the care they receive and raise their satisfaction levels.

In this study, patients with preoperative fear and anxiety had also a significant association with satisfaction with preoperative nursing care. Patients who did not experience preoperative fear and anxiety were found to be 1.01 times more likely to report satisfaction with preoperative nursing care compared to those who did have preoperative fear and anxiety. A similar finding was reported in the study conducted in public hospitals in Addis Ababa [12]. This could be because patients who approach their surgery feeling emotionally stable and at ease may be more receptive to the nursing care they receive. Their ability to maintain composure and relaxation may have a favorable impact on how they view the nursing care they receive, increasing their level of satisfaction. Additionally, patients who do not experience worry or panic before surgery could be better able to express their needs and concerns to the nursing staff. They will be more satisfied as a consequence of this excellent communication, which can improve the standard of care and support they receive. Furthermore, people who are not experiencing preoperative worry or fear may have a more upbeat and hopeful view. This optimistic outlook may lead to a more favorable perception.

Patient expectation of the services was also significantly associated with satisfaction with preoperative nursing care. Participants who had low expectations of hospital services were found to be 3.39 times more likely to express satisfaction with preoperative nursing care compared to those who had high service expectations from the hospital. The possible explanation for this could be that patients who have modest expectations may possess a more pragmatic understanding of the limitations and complexities inherent in the healthcare system. As a consequence, they may display greater gratitude towards the care and attention delivered by the nursing staff, even if it falls short of their initial expectations. Conversely, patients with high service expectations might establish unattainable standards or possess excessively demanding criteria. Consequently, if their expectations are not met, they may experience a sense of disappointment or dissatisfaction with the preoperative nursing care, even if it is of exemplary quality. In contrast, individuals with lower expectations are more likely to find the care they receive to be satisfactory, even if it does not reach the lofty heights of their anticipations.

Likewise, preoperative education was significantly associated with satisfaction with preoperative nursing care. Participants who received preoperative education from nurses were 1.15 times more likely to be satisfied with preoperative nursing care compared to those who did not receive such education from nurses. This finding was similar to the finding of the study conducted at the University of Gondar referral hospital and public hospitals in Addis Ababa [12, 31]. The possible reason for this might be that patients who receive preoperative education from nurses are better prepared for surgery by having knowledge and comprehension of the procedures and expectations surrounding their experience. They feel less nervous and uncertain as a result of this instruction, which may improve how they see the nursing care they get. Preoperative education also increases the likelihood that participants will feel powerful and engaged in their care. They can be more engaged in their healing process and may comprehend the significance of specific nursing interventions. A greater sense of participation and teamwork with the nursing staff may be a factor in increased satisfaction [12].

This study’s results were flavored by Kolcaba’s Comfort Theory, which centers on improving patient satisfaction through attending to their comfort requirements. The study showed that aspects aligning with the theory’s relief component can be improved by meeting particular comfort needs to alleviate pain or discomfort. Additionally, the maintenance of the ease component can be achieved through proactive measures to prevent discomfort to prevent known risk factors that would keep a patient from feeling comfortable, while fulfillment of the transcendence component involves providing patients experiencing physical or emotional discomfort with peace, significance, or opportunities for personal growth through preoperative education and creating a positive nurse-patient relationship through the lens of communication, trust, and empathy in preoperative care.

### Implication of the study

In the context of nursing practice, the findings of this study can help nurses in practice by illuminating the variables influencing patients’ satisfaction with preoperative nursing care. Nurses can create tailored methods of care delivery that improve patient experiences and satisfaction by having a greater understanding of the effects of variables including patient gender, treatment costs, preoperative anxiety, and service expectations. Regarding nursing education, the study emphasizes how crucial it is to include preoperative education and communication skills in nursing curricula. It emphasizes how important it is to give nurses the skills and information they need to properly counsel and assist patients before surgery, allaying their anxieties, controlling expectations, and encouraging favorable patient outcomes. The study establishes the foundation for future research endeavors aimed at delving deeper into the topic of patient satisfaction with preoperative nursing treatment. Additional factors that might affect satisfaction, the efficacy of certain interventions or educational initiatives, and the long-term effects of preoperative nursing care on patient outcomes are all potential areas for further research. This information can support evidence-based procedures and guidelines meant to enhance patients’ overall surgical experiences.

#### Conclusion and recommendation

The study revealed patient satisfaction with preoperative nursing care was high, even though there is room for improvement to ensure optimal healthcare quality. Preoperative care satisfaction is a critical indicator, as even slight deficiencies in this area can have severe consequences, including fatal outcomes. Factors significantly associated with satisfaction in preoperative nursing care were sex, payment status for treatment, preoperative fear and anxiety, patient expectations, and preoperative education.

To address these findings, hospital managers and health policymakers must develop comprehensive strategies aimed at enhancing satisfaction with preoperative nursing care. Initiatives could involve the implementation of tailored training programs for nurses in collaboration with the Ethiopian Federal Ministry of Health, regional health bureaus, and non-governmental organizations. These programs should prioritize equipping nurses with the necessary skills and knowledge to deliver high-quality preoperative care. It is essential to emphasize the need for further research to fully comprehend the specific factors and their impact on patient satisfaction with preoperative nursing care. This research would contribute to a deeper understanding of how nurses can enhance satisfaction levels, ultimately informing the development of evidence-based practices and policies in this crucial healthcare domain.

##### Strength of the study

To enhance the representativeness and generalizability of our study findings, we employed a substantial sample size and incorporated variables that were overlooked in the previous literature. This approach contributes to a more comprehensive understanding of the factors influencing satisfaction with preoperative nursing care and ensures that our findings encompass a wider range of variables, thereby increasing the validity and applicability of the study results.

##### Limitations of the study

It is important to acknowledge that the cross-sectional nature of our study design only allows us to establish associations and correlations between the dependent and independent variables, rather than establishing a cause-and-effect relationship. Furthermore, as the quantitative data were collected through a self-administered questionnaire, there is a possibility of response bias from the respondents, which could introduce some limitations to the validity of the data.

## Data Availability

The datasets generated and/or analyzed during the current study are not publicly available but are available from the corresponding author on reasonable request.

## Declarations

### Disclaimer

We, the undersigned, affirm that this paper is an original piece of work conducted by us and has not been previously presented or published in any Journal. We are fully aware of the importance of academic integrity and understand that plagiarism is strictly prohibited. Any direct quotations or material taken from other sources have been appropriately referenced and credited.

## Abbreviations

AOR: Adjusted Odds Ratio
CI: Confidence Interval
COR: Crude Odds Ratio
OBY/GYN: Obstetrics and Gynecology
SPSS: Statistical Package for Social Sciences
